# Unboxing machine learning models for concrete strength prediction using XAI

**DOI:** 10.1038/s41598-023-47169-7

**Published:** 2023-11-14

**Authors:** Sara Elhishi, Asmaa Mohammed Elashry, Sara El-Metwally

**Affiliations:** 1 Department of Information Systems, Faculty of Computers and Information, Mansoura University, P.O. Box: 35516, Mansoura, 35516 Egypt; 2 Computer Science Department, Faculty of Computers and Information, Mansoura University, Mansoura, 35516 Egypt

**Keywords:** Applied mathematics, Computational science, Computer science, Information technology, Scientific data

## Abstract

Concrete is a cost-effective construction material widely used in various building infrastructure projects. High-performance concrete, characterized by strength and durability, is crucial for structures that must withstand heavy loads and extreme weather conditions. Accurate prediction of concrete strength under different mixtures and loading conditions is essential for optimizing performance, reducing costs, and enhancing safety. Recent advancements in machine learning offer solutions to challenges in structural engineering, including concrete strength prediction. This paper evaluated the performance of eight popular machine learning models, encompassing regression methods such as Linear, Ridge, and LASSO, as well as tree-based models like Decision Trees, Random Forests, XGBoost, SVM, and ANN. The assessment was conducted using a standard dataset comprising 1030 concrete samples. Our experimental results demonstrated that ensemble learning techniques, notably XGBoost, outperformed other algorithms with an R-Square (R^2^) of 0.91 and a Root Mean Squared Error (RMSE) of 4.37. Additionally, we employed the SHAP (SHapley Additive exPlanations) technique to analyze the XGBoost model, providing civil engineers with insights to make informed decisions regarding concrete mix design and construction practices.

## Introduction

Concrete, a widely employed construction material, is renowned for its cost-effectiveness in various infrastructure projects, including but not limited to buildings, bridges, tunnels, and roads. The robustness of these structures, particularly their ability to withstand heavy loads and extreme weather conditions, relies heavily on deploying high-performance concrete with sufficient strength and durability^1,2^. Accurate prediction of concrete strength, accounting for diverse mixtures and varying loading conditions, is paramount for civil engineers. Such predictive capabilities empower engineers to optimize concrete performance, decrease costs, and enhance safety measures^3,4^. The estimation of concrete strength is a process that is typically applied in various phases of civil engineering projects. Initially, during mix design, it is used to ensure the best performance at a minimal cost for the specified project^5^. Subsequently, during construction, physical tests are conducted to verify that the chosen mix meets the desired criteria and performance requirements over time, employing various methods^6^. As a result, concrete strength estimation is a crucial process for performance-based design, optimizing mix proportions, promoting sustainability, facilitating structural health monitoring, and managing risks.

Concrete, a composite material, comprises cement, coarse and fine aggregates (e.g., sand and stone), water, and various admixtures. Accurately forecasting concrete strength within this intricate composition is a significant challenge, given the non-linear relationship between these components and the concrete's strength^7,8^.

Historically, traditional methods have been employed for predicting concrete strength, relying on empirical formulas, analytical models, and physical tests. These physical tests involve the creation of concrete cubes or cylinders according to specified standards, followed by curing and a waiting period until the mixture reaches its maximum strength, typically after 28 days^9,10^. Any errors necessitate repeating the entire process. While these physical tests are straightforward, they are time-consuming, cost-efficient, and prone to providing imprecise results. Some studies have proposed empirical regression methods to calculate proportional ratios based on various elements to predict strength. However, these empirical approaches have struggled to capture the complex, non-linear relationships among different concrete components, making accurate strength prediction challenging^2,4,8,11,12^.

The emergence of Artificial Intelligence (AI) and Machine Learning (ML) techniques has led to solutions to tackle the challenges encountered in structural engineering, including predicting concrete strength^13^. Machine learning models can learn the intricate relationships among various concrete mixture parameters and strengths from historical data, applying this knowledge to make accurate predictions on new data^14–16^. Nonetheless, due to a lack of interpretability and transparency, engineers often find it challenging to comprehend the behavior of machine learning models and the rationale behind their predictions. Introducing the field of Explainable Artificial Intelligence (XAI), which provides the tools to interpret machine learning model predictions, understand their behavior and influencing factors, and analyze the significance of classification features^17–19^.

This paper investigates three machine learning approaches for concrete strength prediction: statistical regression, tree-based ensemble techniques, and artificial neural networks. Regression models aim to establish mathematical functions describing the relationship between input and output variables. While Linear Regression captures simpler relationships, Ridge and Least Absolute Shrinkage and Selection Operator (LASSO) Regression excels at modeling more complex connections. Ensemble learning combines multiple models to enhance prediction accuracy and reduce overfitting. Various models are trained on different subsets of data, with the final prediction being an aggregate of each model's output. Inspired by the structure and functioning of the human brain, Artificial Neural Network (ANN) is introduced as a machine learning model that utilizes the backpropagation technique in the training process to adjust the neurons' weights and minimize errors between predicted and actual outcomes.

To evaluate the performance of these machine learning approaches in predicting concrete strength, we conducted experiments using a real-world dataset containing diverse parameters (e.g., cement, water, coarse aggregate) influencing concrete strength. We employed various evaluation metrics to assess the models’ performance and identify the most accurate and efficient model. Additionally, we explored the impact of XAI techniques on improving transparency and interpretability in concrete strength prediction. The SHapley Additive exPlanations (SHAP) method elucidates factors contributing to the concrete strength prediction^20^. SHAP values are utilized to explain the output predictions of any machine learning model. They employ the concept of game theory to compute the contribution of each 'game player' (feature) to the final 'game outcome' (model prediction). In the context of machine learning, each feature is assigned a value that represents its importance and contribution to the final model prediction. The SHAP method analyzes the effect of each feature, assesses its significance relative to others, and evaluates how features interact within a unified framework to determine the final model prediction output.

The results of our study reveal that ensemble models, specifically XGBoost, surpass regression and ANN models when it comes to predicting concrete strength. Furthermore, the application of XAI techniques substantially improves the transparency and interpretability of machine learning models, offering valuable insights into concrete strength prediction. Engineers can utilize these insights to improve prediction accuracy and enhance the safety of concrete structures.

The paper's structure is as follows: “Related work” reviews previously introduced machine learning models for concrete strength prediction. “Methodology” presents the machine learning framework for concrete strength prediction, encompassing data collection, exploration, preprocessing, model training, testing, evaluation, and explanation stages. “Experimental results” showcases experimental results generated by different models and evaluated using standard metrics. Finally, “Conclusion” concludes the paper, highlights current limitations and outlines future research directions in concrete strength prediction.

## Related work

Recently, there has been a thriving interest in leveraging ML techniques to predict material and structural strength. This section offers a summary of significant research efforts in this field and presents their key contributions in Table 1. Specifically, three ML models, CatBoost, k-Nearest Neighbors, and Support Vector Regression, have been employed to forecast concrete strength^21^. These models are supplied with six features extracted from 249 samples, encompassing parameters such as cement, slag, water, sand, crushed stone, and additives. Among these models, k-Nearest Neighbors has shown the most promising performance, achieving the lowest error rate and the highest determination coefficient.

**Table 1:** Machine learning models and the input features for the concrete strength prediction.

Machine learning models	Input features	Data set size	Best performance	Ref.
CatBoost k-Nearest neighbors Support vector regression	Content of cement, slag, water, sand, crushed stone, and additives	249	k-Nearest neighbors	^21^
Random forests Support vector regression XGBoost	Cement use, age, water, coarse aggregate, fine aggregate, high-efficiency water reducer, fly-ash, and mineral powder	60	XGBoost	^22^
AdaBoost GBDT XGBoost Random forests	Cement, fly-ash, silica fume, coarse aggregate, water, fine aggregate, polypropylene fiber, and a high-performance water reducer	204	GBDT	^23^
Linear regression Classification and regression tree Artificial neural network Support vector machine	Cement, slag, fly-ash, water, superplasticizer, coarse aggregate, fine aggregate, slump test result, and 28-day compressive strength test result	103	Linear regression (lower workability) ANN (higher workability) SVM (higher workability)	^24^
AdaBoost-based model	Cement, water, coarse aggregate, fine aggregate, superplasticizer, blast-furnace slag, fly-ash, curing time	1030	–	^26^
Linear regression LASSO regression Ridge regression Decision trees Random forests XGBoost Support vector machine Artificial neural network	Cement, water, coarse aggregate, fine aggregate, superplasticizer, blast-furnace slag, fly-ash, age	1030	XGBoost	^27^

Another investigation focusing on the prediction of concrete compressive strength has reported that Extreme Gradient Boosting (XGBoost) outperformed Random Forests and Support Vector Regression^22^. This study used eight features, including cement use, age, water content, coarse aggregate, fine aggregate, high-efficiency water reducer, fly-ash, and mineral powder, derived from a dataset comprising 60 samples.

In ensemble learning, an assessment of four models, namely Adaptive Boosting (AdaBoost), Gradient Boosting Decision Trees (GBDT), XGBoost, and Random Forests, was conducted to predict compressive and tensile strength^23^. Based on a dataset consisting of 204 samples, the study employed the eight features outlined in Table 1. The GBDT model exhibited the most promising results among the evaluated models.

To explore the adaptability of concrete properties and compressive strength, another study adopted a combination of four models: Linear Regression, Classification and Regression Tree, ANN, and Support Vector Machine (SVM)^24^. The concrete samples were categorized based on workability, as determined by a slump test with a threshold of 12.5 cm. Linear Regression produced the most accurate predictions for lower workability slump tests, while ANN and SVM excelled in cases of higher workability.

The AdaBoost algorithm has also been applied to forecast concrete compressive strength using a composite of eight components or features alongside information about curing time. Remarkably, this approach outperformed other ML models, including ANN and SVM^7^.

To accurately estimate the compressive strength of fly-ash concrete and reduce the high variance of predictive models, the study in reference^25^ compares various ensemble deep neural network models. These models include the super learner algorithm, simple averaging, weighted averaging, integrated stacking, and separate stacking ensemble models. Among these, the best results, with the highest coefficient of determination, lowest mean squared error, and the highest mean accuracy (97.6%), were achieved using separate stacking with the random forest meta-learner.

Authors of^27^emphasize the importance of establishing a precise model for the bond strength of corroded reinforced concrete, one that minimizes variance and maximizes reliability. In their study, they compared different convolution-based ensemble learning algorithms to determine which ones perform the best. To address these issues, they utilized a database compiled from previous experimental studies on the relative bond strength of corroded reinforced concrete to train convolution-based ensemble learning models. Their results indicate that the convolution-based integrated stacking model provides accurate predictions with coefficients of determination, a-20 index, and mean squared error values of 0.84, 0.75, and 0.022, respectively.

The domain of XAI techniques is becoming increasingly important in the context of predicting concrete strength. These methods assist engineers and researchers in understanding the crucial factors that impact concrete strength, enabling them to make informed decisions regarding concrete mix design and construction practices^28^. Common XAI techniques deployed in concrete strength prediction encompass feature importance analysis at the global and local levels and visual explanations. Feature importance analysis techniques, such as permutation feature importance and SHAP values, are indispensable for discerning the key factors that significantly impact concrete strength prediction. Furthermore, methods for local interpretability, such as LIME (Local Interpretable Model-agnostic Explanations), offer insights into the predictions generated for specific data points^17,29^.

## Methodology

**Figure 1:**
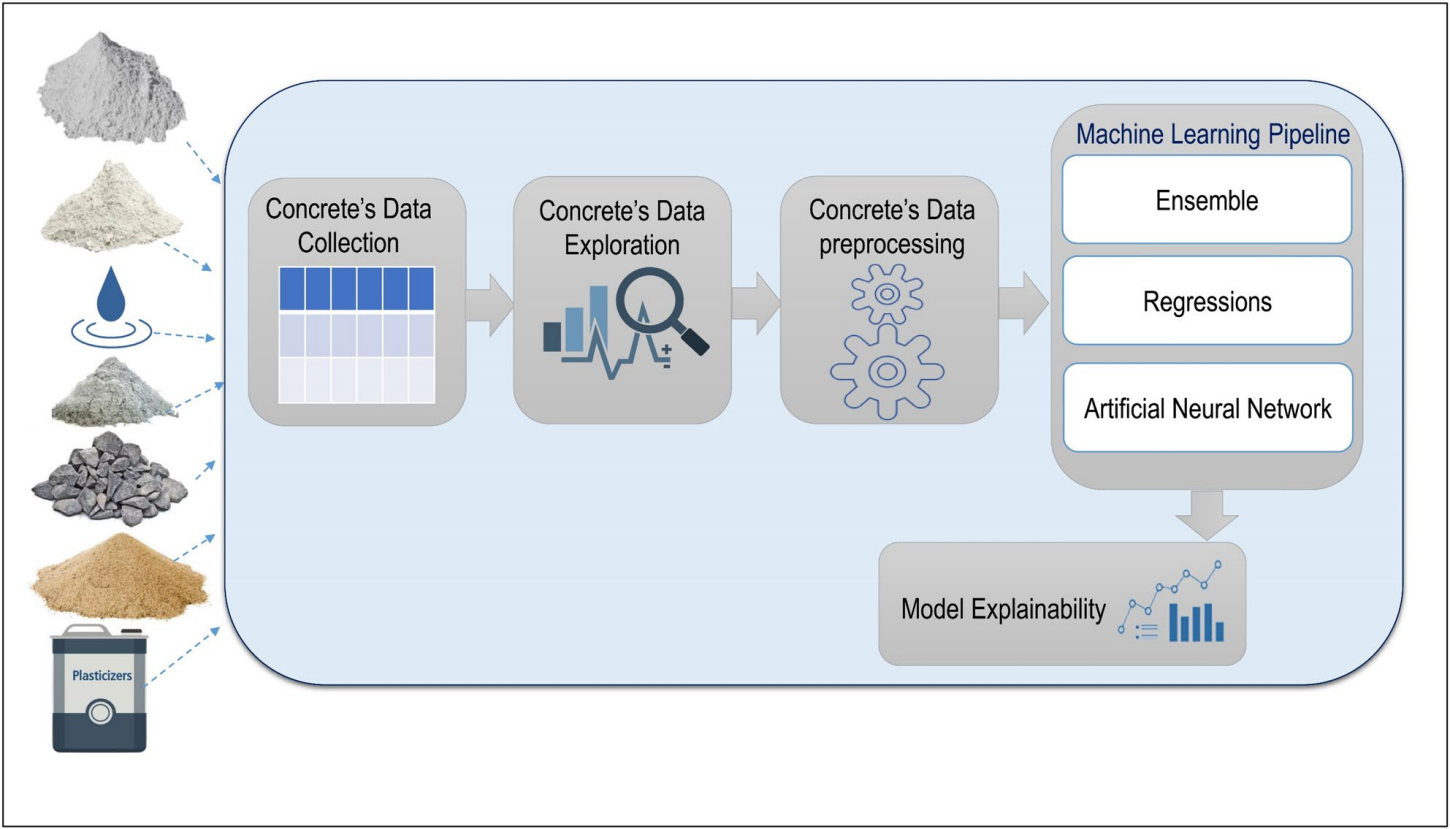
Machine learning pipeline for concrete strength prediction.

The machine learning framework for predicting concrete strength comprises five fundamental stages, as illustrated in Fig. 1. Firstly, the data collection process involves preparing a series of concrete samples under controlled conditions. This includes varying factors such as cement type, water-cement ratio, aggregate size, and curing duration—these represent the input features for the machine learning model. In the next stage, we conduct data exploration to analyze and understand the collected data. The aim is to uncover patterns, relationships, and insights crucial for effectively training machine learning models. Subsequently, the data preprocessing stage is executed to eliminate noise, address missing values, clean the data, and format it appropriately for machine learning model training. We train eight machine learning models, encompassing statistical regression, ensemble learning, SVM, and ANN, to predict concrete strength and these models are evaluated accordingly.

As shown in Table 1, prior research in concrete strength prediction primarily relies on three key approaches: statistical regression techniques, ensemble learning based on Decision Trees, and the utilization of ANNs. Our choice to employ representative machine learning models from these established approaches is rooted in assessing their efficacy in achieving precise and robust predictions. For instance, we incorporate Linear Regression as a fundamental model to serve as a benchmark for regression tasks, owing to its assumption of a linear relationship between input features and the target variable, making it a valuable initial exploration tool. LASSO Regression addresses potential multicollinearity issues within the dataset and enhances model interpretability by penalizing the absolute values of regression coefficients. Ridge Regression, another regularization method, reduces overfitting and enhances model stability by introducing a penalty term to the loss function. Decision Trees are well-known for their proficiency in capturing complex non-linear relationships and interactions among features, making them suitable for datasets characterized by intricate decision boundaries.

We also leverage Random Forests, an ensemble method, to capture complex patterns within the data, which is advantageous for concrete strength prediction. Additionally, we consider XGBoost, an algorithm based on gradient boosting, due to its robustness in handling missing data and outliers, attributes that can greatly enhance concrete strength prediction. SVM is introduced to explore its potential to provide a distinctive perspective on concrete strength prediction, given its effectiveness in managing high-dimensional data and intricate decision boundaries. Lastly, ANNs, known for their capability to decipher intricate data patterns, are integrated into our analysis to investigate whether complex, non-linear models can outperform traditional regression models in predicting concrete strength. A brief comparison of different machine learning approaches for concrete strength prediction is presented in Table 2. Notably, the XGBoost model yields the best-reported results among all eight evaluated models. Further explanation and analysis of its corresponding results are conducted using one of the common XAI techniques, namely the SHAP method. A detailed explanation of each stage in the machine learning pipeline for concrete strength prediction will be provided in the following subsections.

**Table 2:** Brief comparison of different machine learning approaches for the concrete strength prediction.

Approach	Algorithm	Task	Strengths	Weaknesses
Statistical	Linear regression	Fits a linear equation to the data	Simple Interpretable Works well with linear relationships between the features and the target variable	It may not capture complex patterns Sensitive to outliers Prone to overfitting
	LASSO regression	Fits a linear equation to the data with additive regularization (L1) parameter	Prevents overfitting Performs feature selection using the regularization (L1) parameter	Sensitive to data scaling Not ideal for highly correlated features
	Ridge regression	Fits a linear equation to the data with additive regularization (L2) parameter	Prevents overfitting Reduces multicollinearity in the model Better with high-dimensional datasets	Does not perform feature selection Sensitive to feature scaling
	SVM	Maximizes the margin between classes with multiple equations	Effective for high-dimensional data Good at handling imbalanced datasets	Sensitive to parameter settings May require feature scaling
Tree-based	Decision Trees	Divides the data into branches based on feature splits	Strong performance with non-linear relationships Easy to interpret	Prone to overfitting It may create deep trees with high variance
	Random forests	Ensemble version of decision trees	Reduces overfitting Provides feature importance scores	Computationally expensive Less interpretable than a single decision tree
	XGBoost	Uses gradient boosting techniques as a modified version of the Decision tree	High predictive accuracy supports L1 (LASSO) and L2 (Ridge) regularizations Computationally efficient	Sensitive to hyperparameter tuning Less interpretable
Artificial neural network	ANN	A multi-layer network of interconnected nodes (artificial neurons)	Captures complex, non-linear relationships, Scalability and flexibility with data of different sizes	Prone to overfitting Sensitive to feature scaling Sensitive to hyperparameter tuning

### Concrete’s data collection

Data collection involves preparing a series of concrete samples under controlled conditions, varying factors such as cement type, water-cement ratio, aggregate size, curing duration, and other relevant parameters. The concrete specimens are then subjected to compressive strength tests using specialized equipment. The resulting data, which includes the measured compressive strengths, are recorded and used as the basis for training machine learning models. It is crucial to ensure that the data collection follows standardized testing procedures and quality control measures to maintain consistency and reliability. In this paper, the standard dataset collected by Yeh^30^ is used, consisting of 1030 concrete samples with eight features: cement, water, coarse aggregate (coarse), fine aggregate (fine), superplasticizer (sp), blast-furnace slag (slag), and fly-ash (flyash). The sample was treated normally for a while before collecting the data. Then, a conventional compressive test procedure determined the concrete's compressive strength using 150 mm-tall cylindrical specimens.

### Concrete’s data exploration

Concrete strength data exploration involves analyzing and understanding the collected data to uncover patterns, relationships, and insights that can be utilized to train machine learning models effectively. To understand the data distribution and variability, the exploration process started with descriptive statistics, such as mean, median, standard deviation, and quartiles. Visualizations, such as histograms, box plots, and scatter plots, provide further insights by illustrating the relationships between features such as cement, water, coarse aggregate, and fine aggregate and identifying potential outliers or anomalies.

### Concrete’s data preprocessing

Data preprocessing is crucial in accurately predicting concrete strength using machine learning techniques. In concrete strength prediction, data preprocessing involves several important steps. Firstly, data cleaning is performed to handle missing values, outliers, and inconsistencies in the dataset. Missing values can be imputed using appropriate mean or median imputation techniques. Outliers can be detected and treated by removing or replacing them with more representative values. Data cleaning is essential to ensure the quality and integrity of the dataset. Handling missing values, outliers, and inconsistencies is crucial because these issues can introduce noise and inaccuracies into the model. For instance, missing values in features related to concrete composition can lead to biased predictions if not appropriately imputed. Outliers, such as extreme strength values, can skew the model's understanding of the typical concrete properties. By addressing these issues, we enhance the reliability of our predictive model. Secondly, feature scaling is applied to ensure that all features are on a similar scale. Common techniques include normalization or standardization, which is necessary because concrete-related features often have different units and scales. For example, the compressive strength of concrete might be measured in MegaPascals (MPa), while the curing time is measured in days. If these features are not scaled, those with larger magnitudes could dominate the learning process of machine learning algorithms. Normalization or standardization ensures that all features contribute more equally to the prediction, preventing any one feature from excessively influencing the model's output. In concrete strength prediction, we often encounter categorical variables like the type of cement used or the curing method applied. Machine learning models require numerical input, so we encode these categorical variables into numerical values. One-hot encoding or label encoding helps to represent categorical data in a format that the algorithms can work with. By performing these preprocessing steps, the dataset is appropriately prepared, enhancing the effectiveness and performance of machine learning algorithms in predicting concrete strength.

### Machine learning approaches for concrete strength prediction

**Figure 2:**
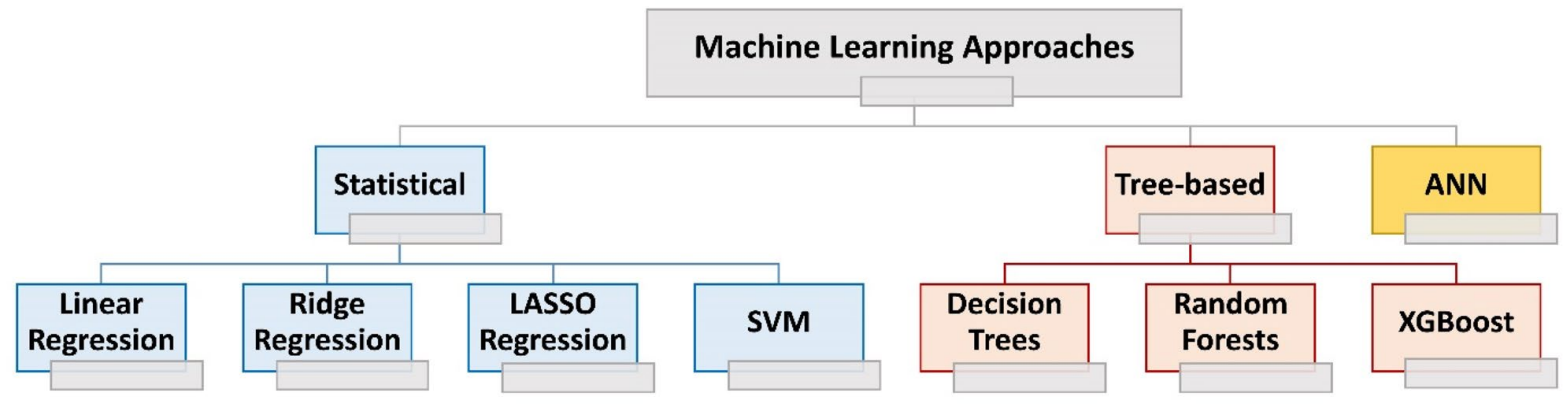
Three machine learning approaches for concrete strength prediction.

This paper studies three machine learning approaches in concrete strength prediction: statistical, tree-based learning, and ANNs techniques (see Fig. 2). The statistical learning methods are simple predictive techniques used to identify the relationship between the input and output variables and develop a mathematical function that accurately describes this relationship. Statistical learning models include Linear Regression, Ridge Regression, LASSO Regression, and SVM. Tree-based learning methods are supervised machine learning techniques that use Decision Trees as the fundamental building blocks for constructing predictive models. The input data is represented as a set of feature vectors, where each vector contains a set of predictor variables (features) and the corresponding target variable. The goal is to learn a mapping between the input features and the target variable. Tree-based learning models include Decision Trees, Random Forests, and XGBoost. The third machine learning approach is based on the ANNs, which are computational models inspired by the structure and functioning of biological neural networks in the human brain. The network consists of interconnected nodes, or neurons, organized in layers. Each neuron receives inputs, performs a weighted computation, and passes the output through an activation function. During training, the network adjusts the weights associated with each connection to minimize the prediction error. Once trained, the ANN can take new inputs and provide predictions of concrete strength based on the learned patterns and relationships in the training data. The details explanation of each approach is presented in the following subsections.

#### Statistical machine learning approaches

The statistical learning methods are simple predictive techniques used to identify the relationship between the input and output variables and develop a mathematical function that accurately describes this relationship. Statistical learning models include linear Regression, Ridge Regression, LASSO Regression, and SVM. Linear regression as a statistical model assumes a linear relationship between one (or more) independent variables ***X*** and specific dependent variable ***y***. This linear relationship can be expressed using a simple line equation with the perfect coefficient ***β*** (or coefficients), such that

1 $$y={\beta }_{0}{x}_{0} +{\beta }_{1}{x}_{1} +.....+{\beta }_{n}{x}_{n}$$

where, X = [${x}_{0}, {x}_{1}, ... , {x}_{n}$] and ***β* = **[${\beta }_{0}, {\beta }_{1}, ... , {\beta }_{n}$] represented as vectors with $x_{0} = 1$.

With basic Linear Regression, to estimate the best set of feature coefficients ***β***, the error between actual and predicted values for ***y*** must be decreased to the minimum^5^. This error is calculated by the least squares method such that the sum of squares (SSE) is minimized.

2 $$SSE=\sum_{i=0}^{N}{\left({y}_{i}-{\widehat{y}}_{i}\right)}^{2 }$$

$${\mathrm{and }\widehat{y}}_{i}= {\beta }_{i0}{x}_{i0} +{\beta }_{i1}{x}_{i1} +.....+{\beta }_{in}{x}_{in}$$

where ${y}_{i}$ and ${\widehat{y}}_{i}$ are the actual and predicted values for the *i*^th^ record. The minimization of SSE can be achieved with different methods^31^.

Ridge regression was introduced by Hoerl and Kennard^32^ to improve prediction accuracy while dealing with highly correlated features. It shrinks the regression coefficients by adding a penalty on their size depending on the ridge coefficient. The error between the actual and predicted value is,

3 $$Ridge \,\,Error=\sum_{i=0}^{N}{\left({y}_{i}-{\widehat{y}}_{i}\right)}^{2 }- \lambda \sum_{J=1}^{P}{\beta }_{j}^{2}$$

Lambda $\lambda$ is a complexity parameter that controls the amount of shrinkage, such as increasing lambda produces greater shrinkage.

LASSO Regression is another shrinkage method like ridge but uses the absolute values coefficients ***β*** while calculating the error function as follows:

4 $$LASSO \,\,Error =\left(\frac{1}{2}\sum_{i=0}^{N}{\left({y}_{i}-{\widehat{y}}_{i}\right)}^{2 }-\lambda \sum_{J=1}^{P}|{\beta }_{j}|\right)$$

Replacing the Ridge penalty term $\lambda \sum_{J=1}^{P}{\beta }_{j}^{2}$ by the LASSO penalty term $\lambda \sum_{J=1}^{P}|{\beta }_{j}|)$ makes the solutions nonlinear in the ${y}_{i}$ and there is no closed-form expression as in ridge regression. In some cases, data cannot be linearly separable, where different classes can overlap. In this case, separating classes with a single line is impossible. The main function of SVM is to form a hyperplane and a decision boundary using this plain defined by a set of support points to separate different classes in data^33^.

#### Tree-based machine learning approaches

Tree-based learning methods are supervised machine learning techniques that use decision trees as the fundamental building blocks for constructing predictive models. The input data is represented as a set of feature vectors, where each vector contains a set of predictor variables (features) and the corresponding target variable (the variable we want to predict). The goal is to learn a mapping between the input features and the target variable. Examples of Tree-based learning models are Decision Trees, Random Forests and XGBoost. Instead of working with the whole dataset, Decision Trees split the data among set decisions according to specific values, constructing a Tree Structure. The tree continues splitting to generalize the final decision over the whole data. A tree size parameter should be adaptively chosen from the data to control this continuous splitting. Different methodologies can be used to choose the optimal tree size, including the Sum of Squares (SSE) with a threshold or cost-complexity pruning^33^. While Decision Trees build only one tree structure for data and give the output according to it, Random Forests^34^ build multiple Trees over the same dataset and collect the average output from these trees to give the final decision about specific data records. Random Forests solve the problem of overfitting that the traditional Decision Trees can produce. It also can be affected by the characteristics of data. Gradient Boosting is used with many learning methods^35,36^, but XGBoost is a scalable machine learning system used for tree boosting as an open-source package^37^. It deals with sparse data using its tree learning algorithm besides parallel and distributed computing strategies that speed up the learning process.

#### Artificial neural network machine learning approach

**Figure 3:**
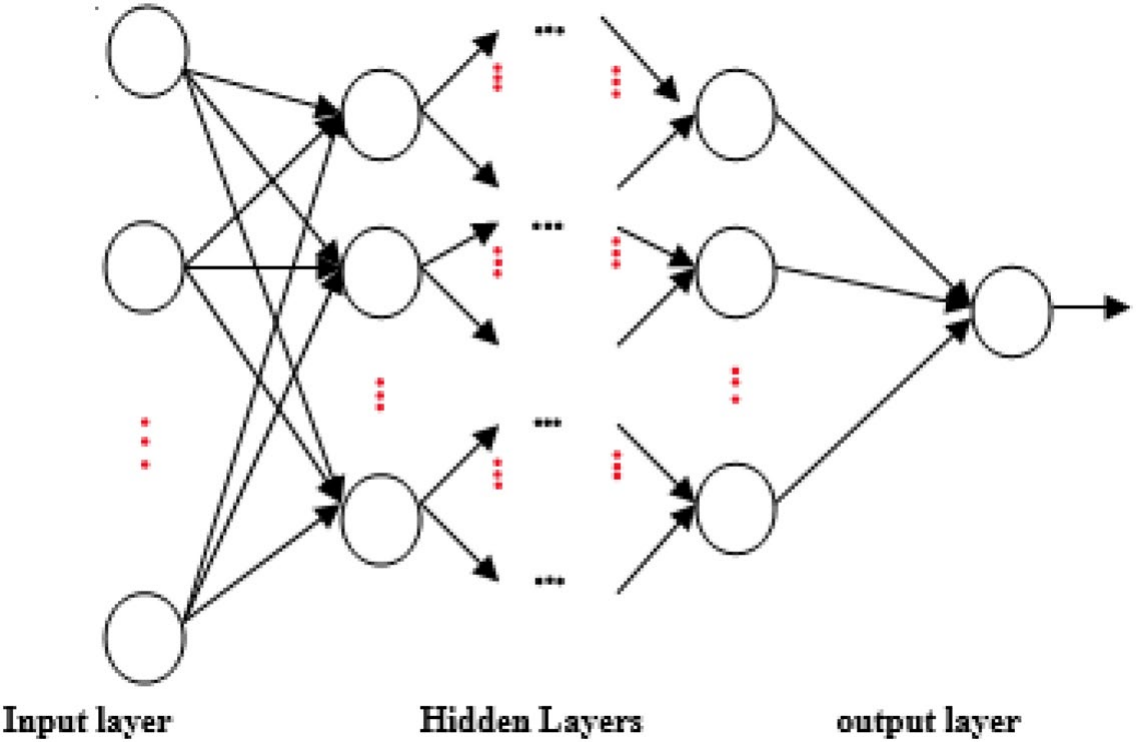
Artificial neural network topology.

Artificial Neural network is a way to mimic human brains, with their tiny components as neurons. In computer science, a neuron is a processing unit represented as a node in a big network. This node can take input value(s) multiplied by a weight parameter, implement a definite process called activation function, and deliver the output(s) to the next neuron(s) or as a final output, as shown in Fig. 3. The topology for a simple neural network is shown in Fig. 2. Where the network consists of a set of layers, each layer has a predefined number of neurons. The main layers are the input, hidden, and output layers. In most cases, the number of neurons in the input layer is identical to the number of features in the dataset, and the output layer gives the predicted value for a specific instance in data^9^.

### Concrete strength prediction model explainability

XAI techniques are becoming increasingly important in concrete strength prediction by helping engineers and researchers understand the key factors contributing to concrete strength and making informed decisions about concrete mix design and construction practices. We used the SHAP method of the XAI techniques to provide insights into the factors contributing to the concrete strength prediction problem^20^. SHAP provides a unified framework that combines game theory and machine learning to attribute the concrete strength prediction outcome to input features such as cement, water, coarse aggregate, fine aggregate, superplasticizer, blast-furnace slag, and fly-ash. By calculating SHAP values, the importance of each feature in contributing to the prediction can be quantified^38^. These values capture the additive contribution of a feature across all possible feature subsets, considering the interactions and dependencies among them. SHAP values help reveal the relative influence of features on the model's output, enabling a deeper understanding of the underlying mechanisms. In the context of AI explainability for concrete strength prediction, SHAP values identify which features play the most significant role in determining the predicted strength. This knowledge allows engineers and researchers to interpret the model's decision, validate reliability, detect biases, and gain insights into improving concrete mix designs and related factors. SHAP values provide transparency and accountability, helping to build trust in AI models and facilitating informed decision-making in concrete strength prediction and other applications.

**Figure 4:**
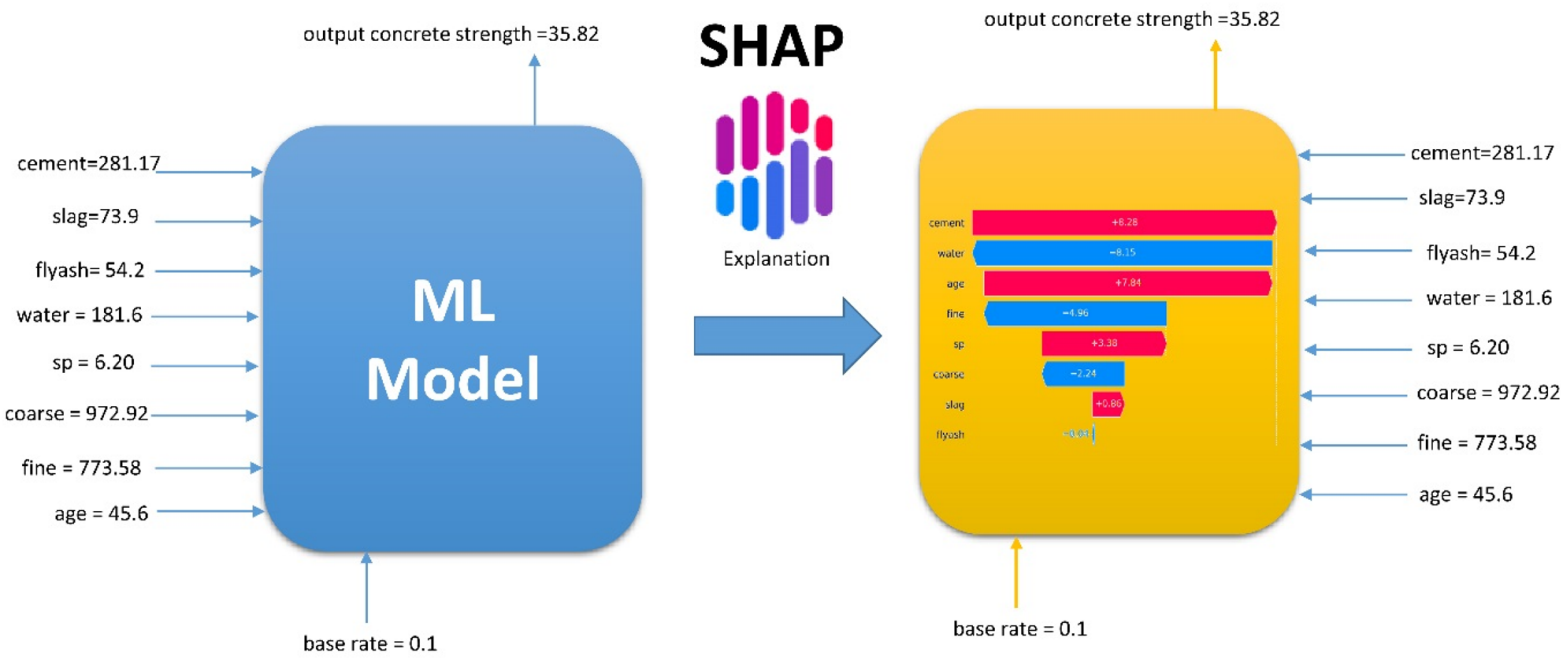
Feature contributions using SHAP for concrete strength prediction.

Figure 4 displays the potential features for the concrete prediction machine learning model, along with the explanation process using the SHAP method, which illustrates the feature values contributing to shifting the model's prediction output from the baseline value. The baseline, in this context, represents the average model output over the training observations on which the model was trained. Features that push the model's predictions higher are color-coded in red, while those causing predictions to decrease are represented in blue.

## Experimental results

### Concrete data collection and exploration

For training and validating machine learning algorithms, this study employs a benchmark dataset made up of 1030 concrete tests with the following eight features: cement, water, coarse aggregate (coarse), fine aggregate (fine), superplasticizer (sp), blast-furnace slag (slag), fly-ash (flyash), and age. A sample from dataset records is presented in Table 3.

**Table 3:** Sample records for concrete strength prediction in this study.

	Cement (m^3^)	Slag (kg/m^3^)	Flyash (kg/m^3^)	Water (m^3^)	sp (%)	Coarse (kg/m^3^)	Fine (mm)	Age (days)	Concrete strength (psi)
0	540	0	0	162	2.5	1040	676	28	79.99
1	540	0	0	162	2.5	1055	676	28	61.89
2	332.5	142.5	0	228	0	932	594	270	40.27
3	332.5	142.5	0	228	0	932	594	365	41.05
4	198.6	132.4	0	192	0	978.4	825.5	360	44.3

**Figure 5:**
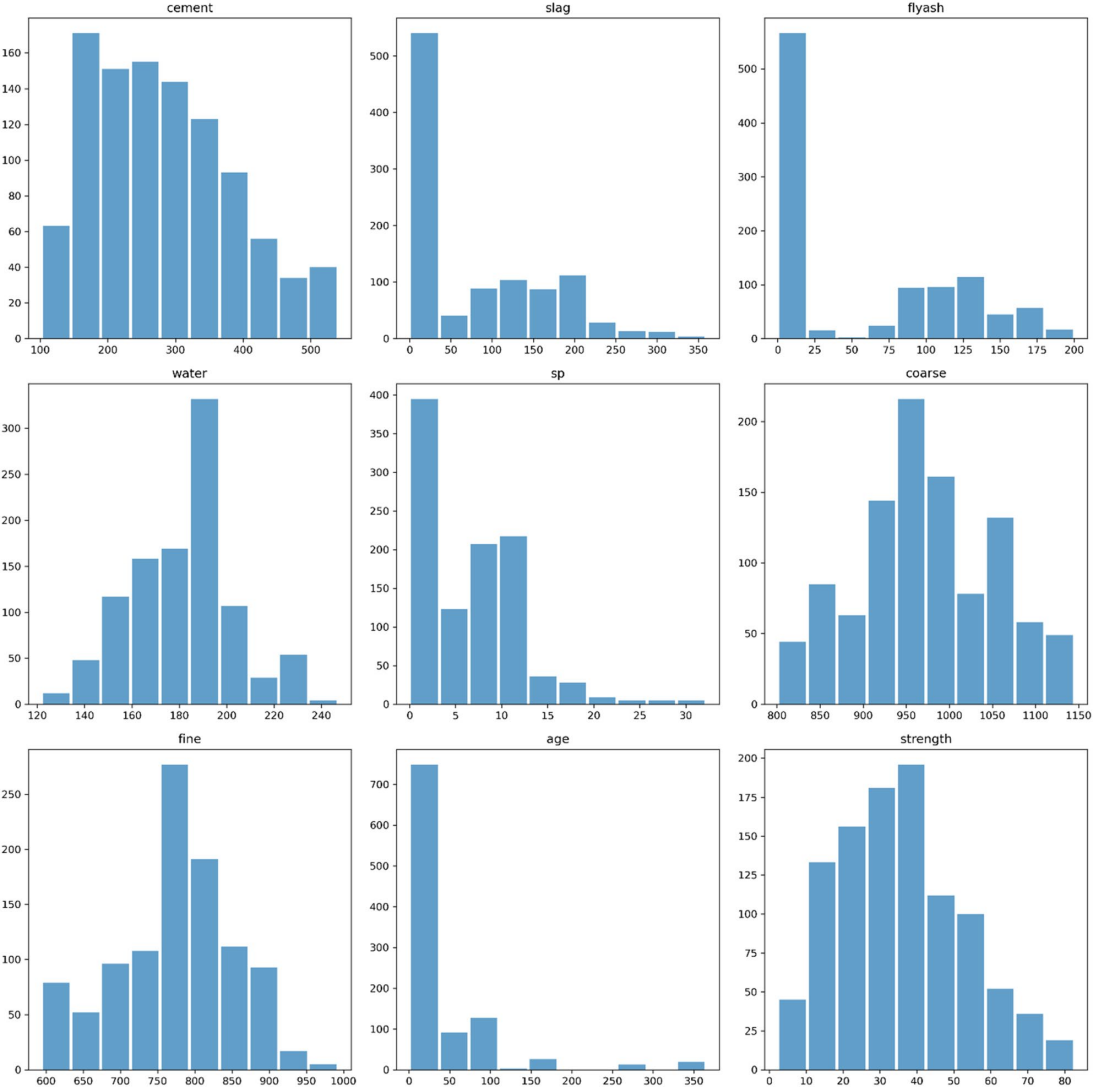
Concrete data exploration process (statistical features distribution).

The min/max values, mean value, standard deviation (std), and quartile distribution are presented in Table 4 as a summary of these attributes and the data exploration procedure for them. Additionally, Fig. 5 displays histograms representing the statistical distribution of relevant features. The x-axis corresponds to each feature, and the y-axis indicates the frequency of occurrences. This visualization allows for a comprehensive evaluation of these features.

**Table 4:** Concrete data exploration process (features summarization).

	Cement (m^3^)	Slag (kg/m^3^)	Flyash (kg/m^3^)	Water (m^3^)	sp (%)	Coarse (kg/m^3^)	Fine (mm)	Age (days)	Concret strength (psi)
Count	1030	1030	1030	1030	1030	1030	1030	1030	1030
Mean	281.16786	73.89583	54.18835	181.56728	6.20466	972.91893	773.58049	45.66214	35.817961
Std	104.50636	86.27934	63.997	21.354219	5.973841	77.753954	80.17598	63.16991	16.705742
Min	102	0	0	121.8	0	801	594	1	2.33
25%	192.375	0	0	164.9	0	932	730.95	7	23.71
50%	272.9	22	0	185	6.4	968	779.5	28	34.445
75%	350	142.95	118.3	192	10.2	1029.4	824	56	46.135
Max	540	359.4	200.1	247	32.2	1145	992.6	365	82.6

Notable observations include:

- cement exhibits a distribution that closely resembles a normal distribution.
- blast-furnace slag (abbreviated as 'slag') displays a proper skewness and appears to follow a distribution with three peaks (Gaussians).
- fly-ash (abbreviated as 'flyash') is right-skewed and appears to have a bimodal distribution with two peaks (Gaussians).
- water's distribution shows three peaks with a leftward tilt.
- superplasticizer (abbreviated as 'sp’) demonstrates a distribution with two peaks and proper skewness.
- coarse aggregate's (abbreviated as 'coarse') distribution is close to normal and displays three peaks (Gaussians).
- fine aggregate's (abbreviated as 'fine') distribution appears to be bimodal with two peaks, indicating a non-normal distribution.
- The age feature appears to have multiple peaks and a skewed distribution, which may be appropriate for the dataset.

**Figure 6:**
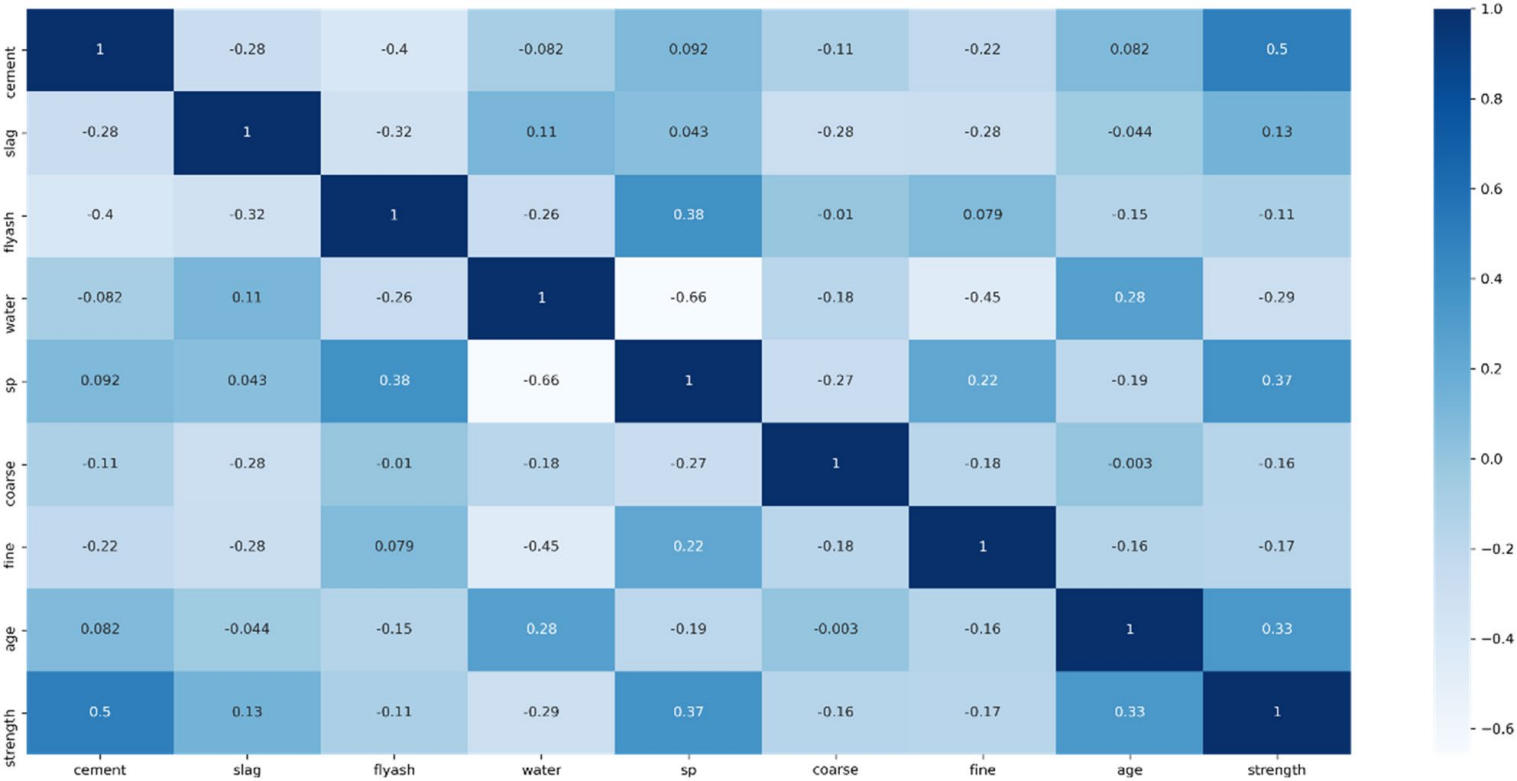
Concrete data exploration process (features correlation).

Studying the correlation between features is essential for understanding the relationships between dependent features and the target strength factor, as this analysis aims to identify the optimal prediction model. Figure 6 displays a heatmap illustrating each variable's impact on all other variables. Notably, a strong correlation is observed between cement and strength, indicating that cement is a highly reliable predictor. Conversely, slag and fly-ash show weak correlations with the target variable. Additionally, it is worth highlighting the significant positive correlation between superplasticizer and fly-ash, in contrast to the comparatively weaker correlation between superplasticizer and compressive strength. Remarkably, there is a substantial negative correlation between water and superplasticizers, as well as between water and strength.

**Figure 7:**
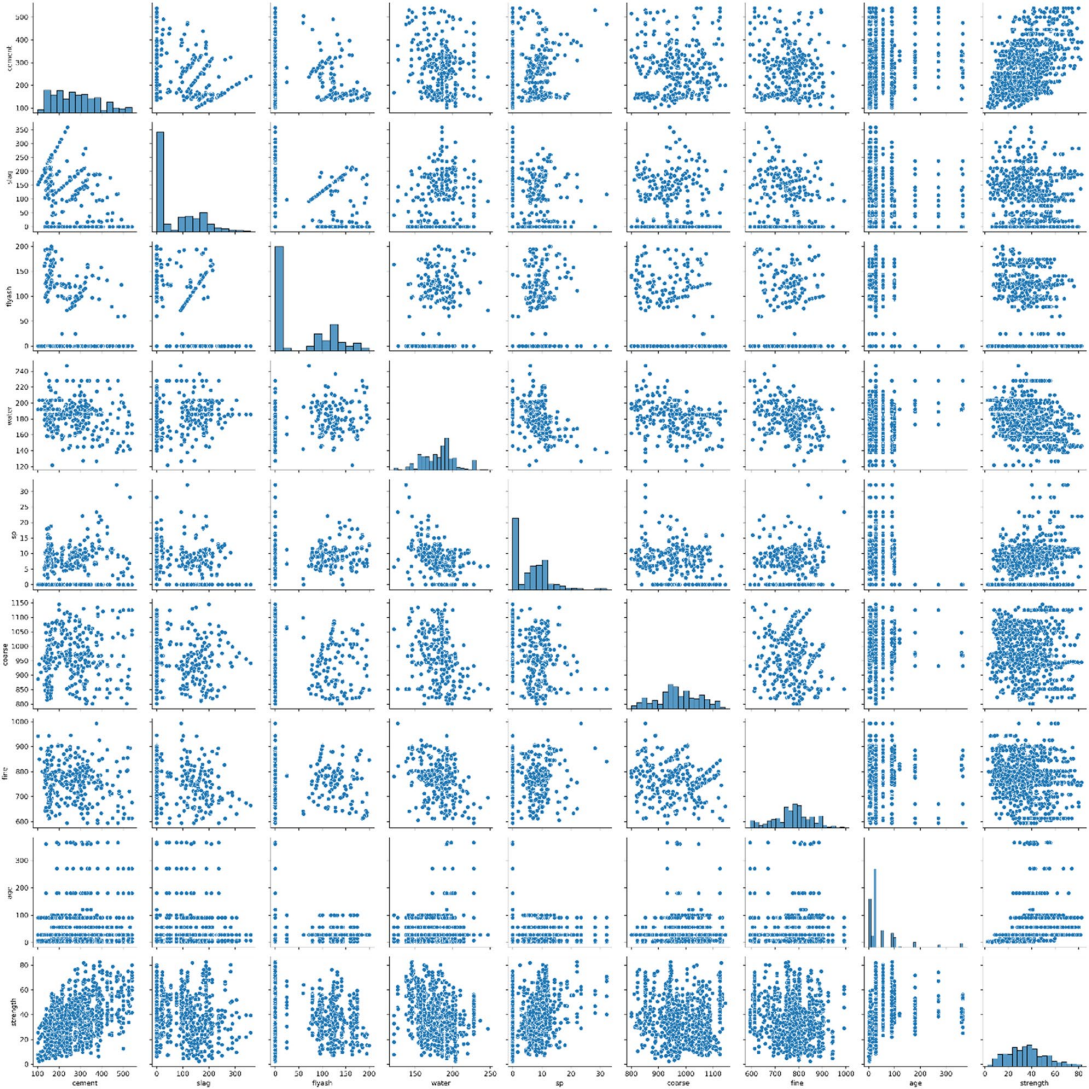
Concrete data exploration process (features correlation pair plot).

Figure 7, presented as a Pair Plot, also visually conveys the features' correlation information. This comprehensive analysis is important because dimensions showing strong correlations with values near 1 or -1 are redundant, supplying the model with duplicated information. As a result, we could decide to keep one dimension while discarding another. The choice of which dimension to retain and which to discard relies on domain expertise and an assessment of which dimension is more error-prone.

From the pair plot, it becomes evident that:

- Cement exhibits complete lack of correlation with other characteristics, including slag, fly-ash, water, superplasticizer, coarse aggregate, fine aggregate, and age.
- Slag also demonstrates no correlation with the following characteristics: fly-ash, water, superplasticizer, coarse aggregate, fine aggregate, and age.
- Fly-ash, aside from lacking any significant correlation with water, superplasticizer, coarse aggregate, fine aggregate, or age, does not exhibit substantial correlation with any other independent attributes.
- In terms of water's relationship with other independent characteristics, a negative linear association is observed with both superplasticizer and fine aggregate. It does not show a meaningful correlation with any other attributes. It is noteworthy that superplasticizers can reduce water content in concrete by 30% without compromising workability.
- Superplasticizer demonstrates a negative linear relationship solely with water and does not exhibit a strong correlation with any other variables.
- Coarse aggregate, like all other attributes, does not display significant correlation with any other attributes.
- Fine aggregate, when compared to unrelated variables, exhibits a linear inverse relationship with water and does not show any meaningful correlation with other characteristics.

**Figure 8:**
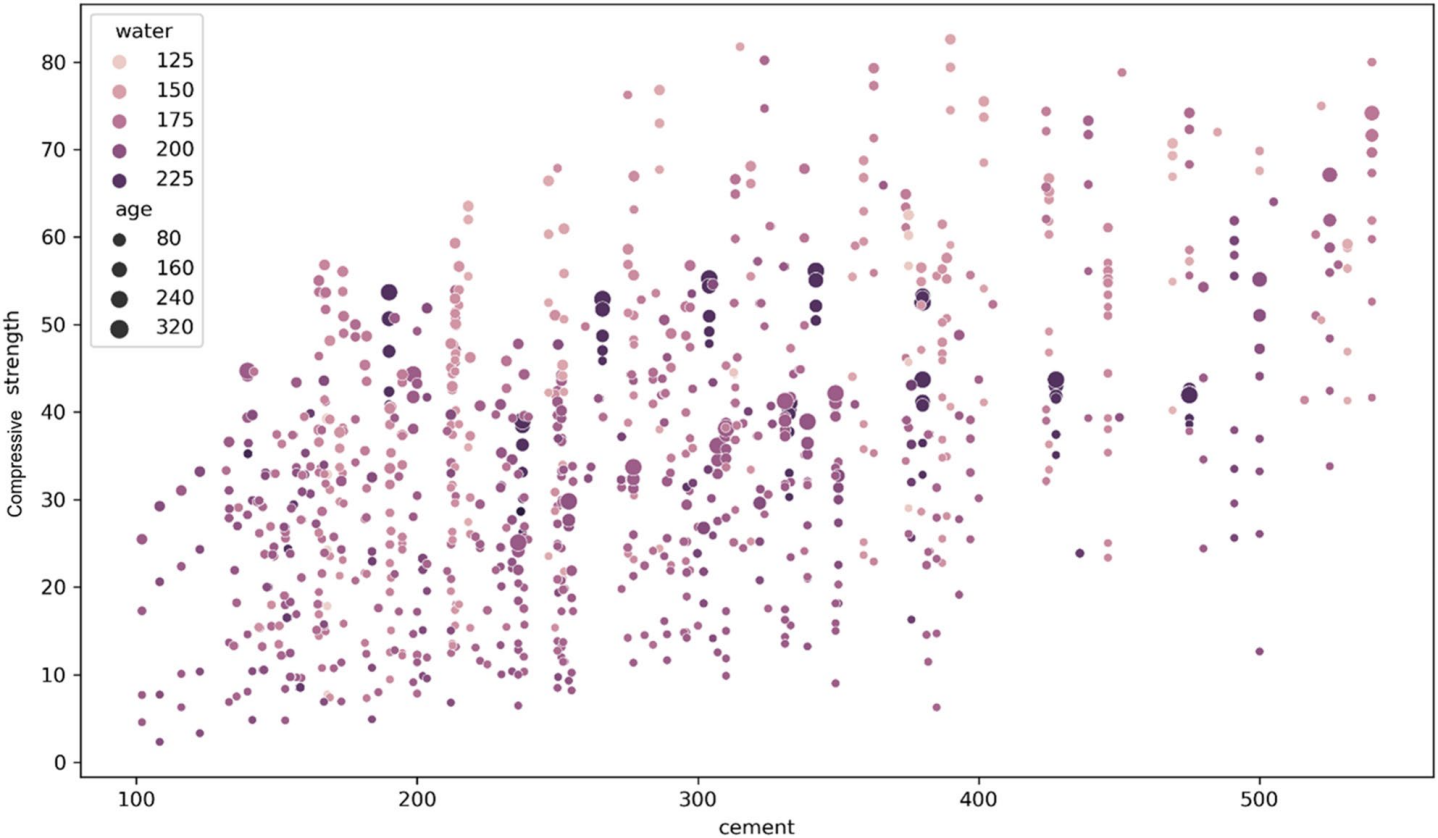
Scatter plot for visualizing the cement and compressive strength relationship.

**Figure 9:**
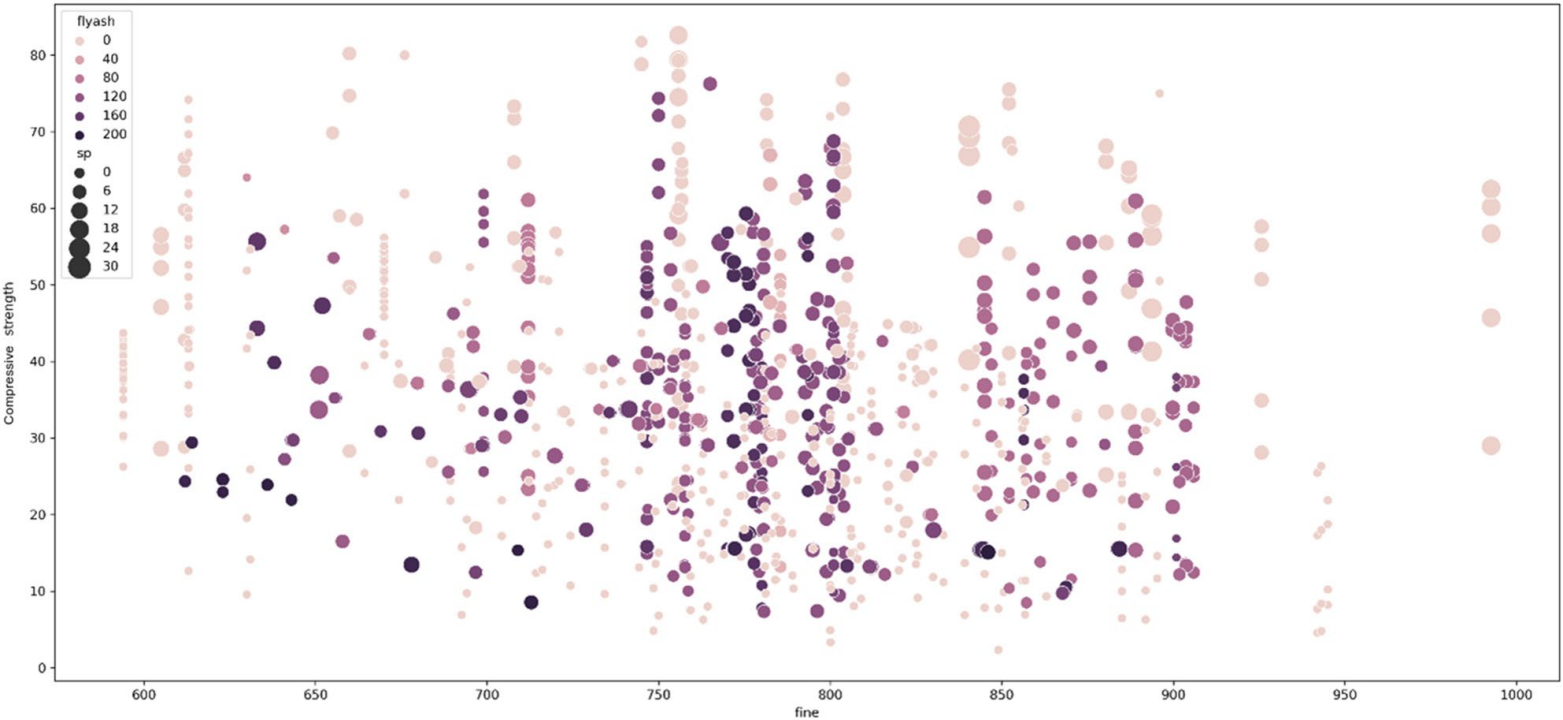
Scatter plot for visualizing the fine-aggregate and compressive strength relationship.

Figures 8 and 9 depict the scatter plots that illustrate the relationship between compressive strength as the target predicted variable and the input features variables cement, water, age, and fly-ash. Figure 8 demonstrates a positive correlation between cement content and compressive strength. As the amount of cement used increases, the compressive strength of the concrete also increases. Moreover, it has been observed that as concrete ages, its strength grows, requiring more cement to achieve greater strength at a younger age. Conversely, older cement necessitates more water, so reducing the water content in concrete enhances strength. The scatter plot in Fig. 9 reveals an inverse relationship between compressive strength and fly-ash content. The concentration of darker dots in the region corresponding to lower compressive strength values makes this relationship evident. Conversely, it has been demonstrated that the use of a superplasticizer improves compressive strength, highlighting a positive association between these two parameters.

### Concrete data preprocessing

The dataset was split into a training set, consisting of 80% of the entire dataset, and a test set, comprising the remaining 20%. The training dataset was used to evaluate the model's structure and parameters. By assessing the performance of different models on the training dataset, we can determine which model has been appropriately trained. However, it is important to note that the test dataset is only employed to assess the effectiveness of the selected model after it has been chosen. Subsequently, the data underwent normalization using a standard scalar. Given that the dataset contains multiple variables, the objective was to rescale all features such that they have a mean of zero and a standard deviation of 1. This normalization process ensures that all features are on a comparable scale, preventing any individual feature from dominating the model's learning process.

### Machine learning models evaluation

Eight machine learning models from three different approaches are evaluated in the presented study. These models include Linear Regression, Ridge Regression, LASSO Regression, SVM, Random Forests, Decision Trees, XGBoost, and ANN. The evaluation is performed using training and testing datasets, and various metrics are applied, such as Mean Squared Error (MSE), Root Mean Square Error (RMSE), Mean Absolute Error, and the coefficient of determination known as R-squared (R^2^) score. MSE, RMSE, and MAE depend on the actual data values and the predictions made by the machine learning models, while the R-squared score is based on data variance. The following are the statistical equations that describe these evaluation metrics:

5 $$\mathrm{MSE}= \frac{1}{N}\sum_{i=0}^{N}{\left({y}_{i}-{\widehat{y}}_{i}\right)}^{2 }$$

6 $$\mathrm{RMSE}= \sqrt{\frac{1}{N}\sum_{i=0}^{N}{\left({y}_{i}-{\widehat{y}}_{i}\right)}^{2 }} $$

7 $$MAE=\frac{1}{N}\sum_{i=0}^{N}\left|\left({y}_{i}-{\widehat{y}}_{i}\right)\right.| $$

8 $${R}^{2} = \frac{\sum_{i=0}^{N}{\left({\widehat{y}}_{i}-{\overline{Y} }_{i}\right)}^{2 }}{\sum_{i=0}^{N}{\left({y}_{i}-{\overline{Y} }_{i}\right)}^{2 }}$$

While ${y}_{i}$ is the actual value, ${\widehat{y}}_{i}$ is the predicted value and ${\overline{Y} }_{i}$ is the mean value of the actual values in data.

The outcomes of various statistical tests conducted by the models on the dataset, as per the expected values, are presented in Table 5. These results demonstrate the successful prediction of concrete compressive strength by all models, as indicated by the statistical performance metrics. Among the models, XGBoost achieved the highest R^2^ value (R^2^ = 0.91), making it the most accurate. Following closely, the Random Forests model obtained an R^2^ value of 0.89, while Decision Trees achieved 0.82, and ANN attained 0.74. In contrast, the Linear Regression models exhibited lower accuracy, with the basic model achieving an R^2^ value of 0.57, followed by LASSO Regression (R^2^ = 0.54), Ridge Regression (R^2^ = 0.57), and SVM (R^2^ = 0.66).

**Table 5:** Benchmarking of eight machine learning models for concrete strength prediction.

Machine learning approaches	Method	RMSE	MSE	MAE	R^2^
Statistical	Linear regression	10.28	105.76	8.23	0.57
	LASSO regression	10.68	114.11	8.65	0.54
	Ridge regression	10.29	105.84	8.24	0.57
	SVM	9.13	83.39	7.44	0.66
Tree-based	Decision trees	6.65	44.24	4.47	0.82
	Random forests	5.21	27.17	3.53	0.89
	XGBoost	**4.37**	22.33	3.04	**0.91**
Artificial neural network	ANN	6.01	36.22	4.53	74.63

Significant values are in bold.

**Figure 10:**
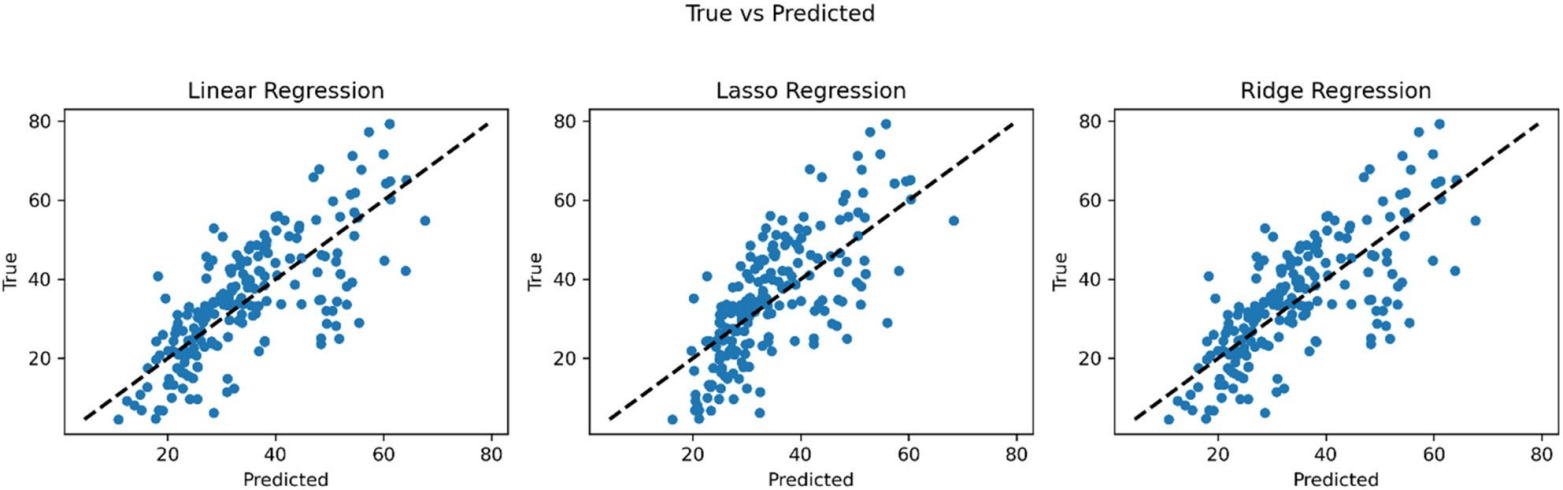
Linear, ridge, and LASSO regression models scatter plots.

**Figure 11:**
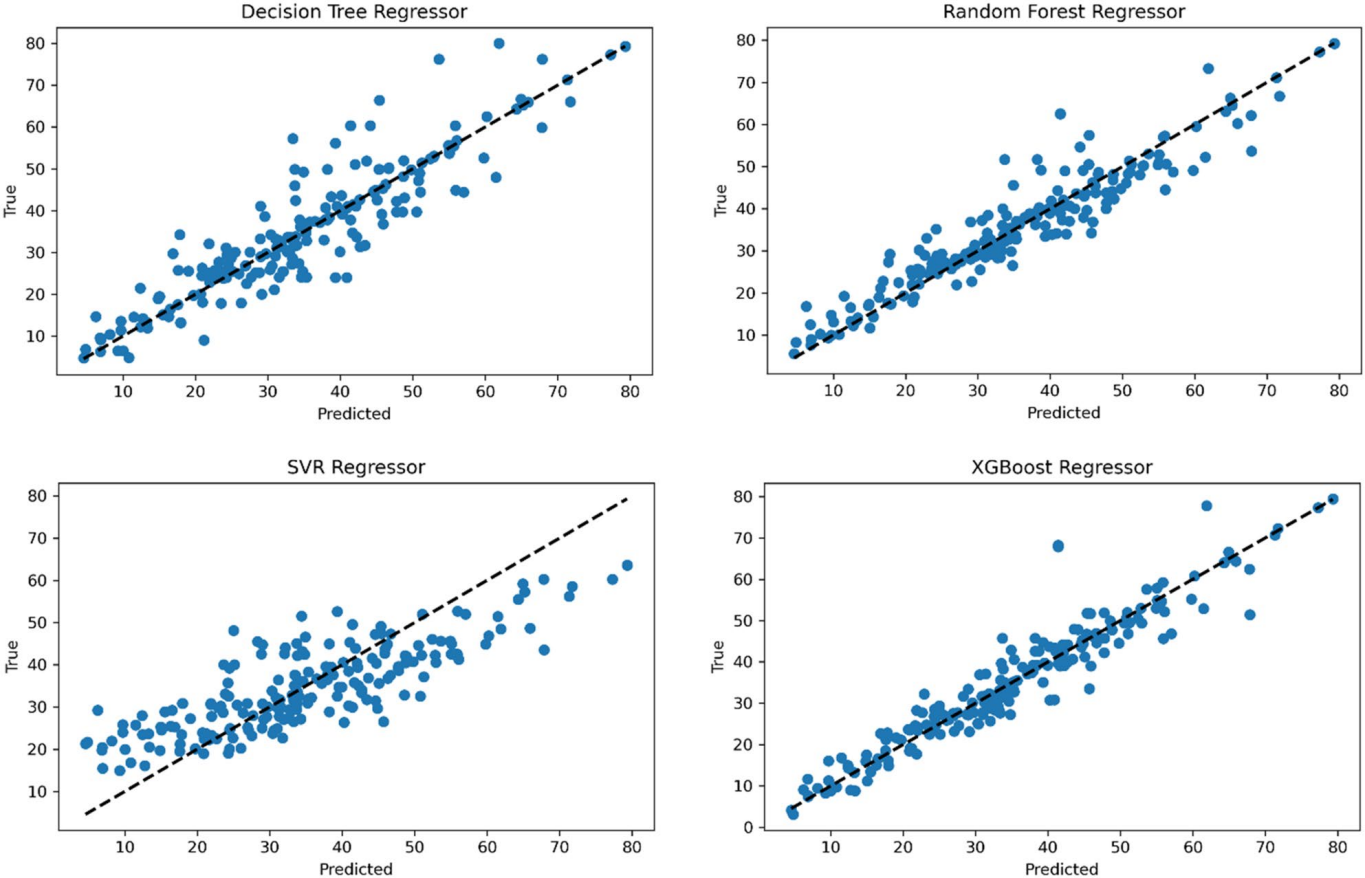
Decision trees, random forests, SVM, and XGBoost models scatter plots.

Regarding the statistical error, it is noteworthy that the XGBoost model recorded the lowest RMSE value (4.37), while the SVM model and Regression models displayed higher values, with SVM having an RMSE of 9.13 and Regression models averaging around 10. Based on the accuracy criterion, it can be concluded that XGBoost is currently the best-performing model. The scatter plots of the experimental (actual) and predicted compressive strengths of concrete are depicted in Figs. 10 and 11, respectively.

**Figure 12:**
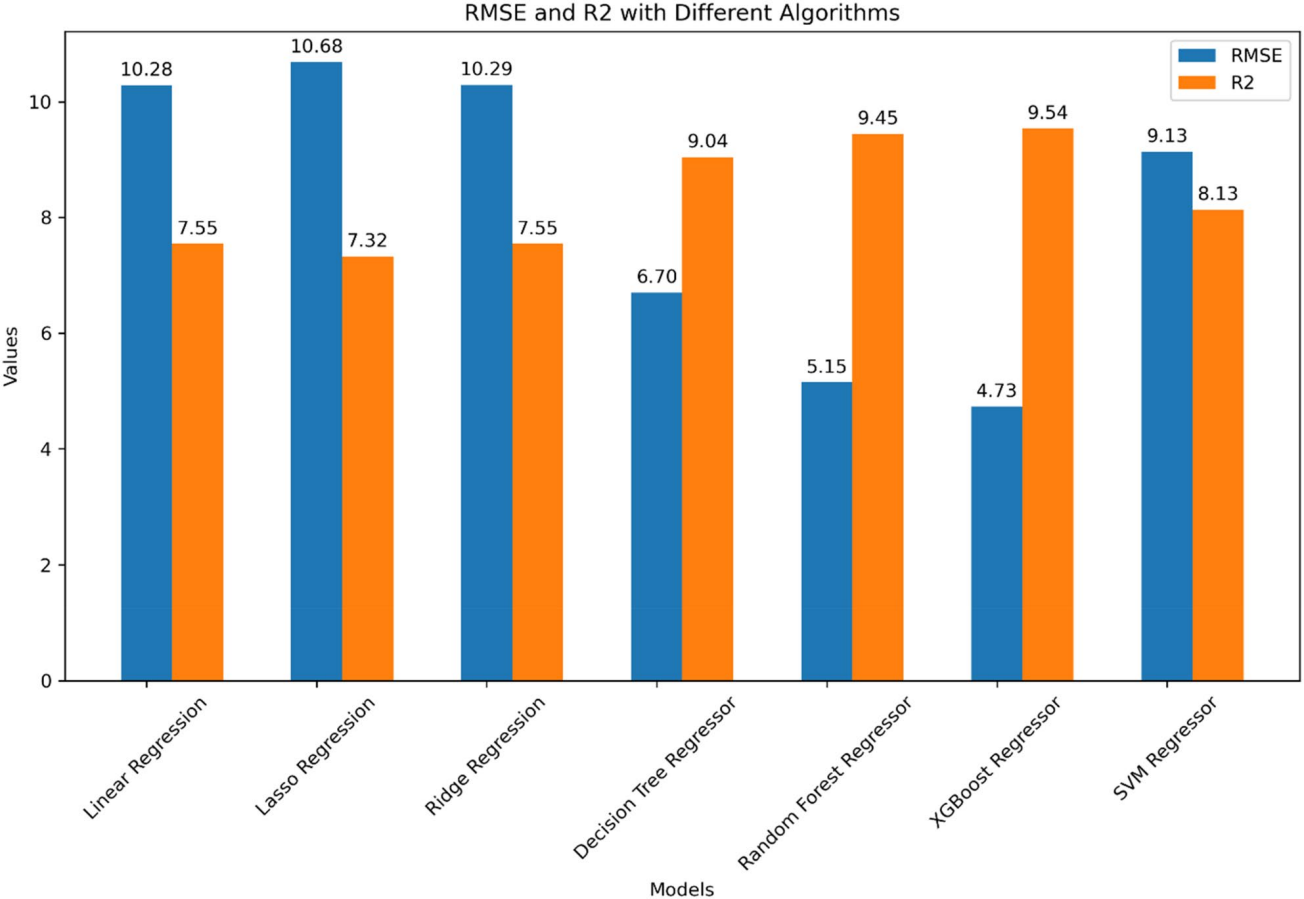
Concrete strength prediction machine learning model’s RMSE and R^2^ evaluation metrics.

The R^2^ and RMSE values from the seven models are shown in Fig. 12. With the input feature variables: cement, water, coarse aggregate, fine aggregate, superplasticizer, blast-furnace slag, and fly-ash, this figure implies that the XGBoost model could be effective and have tolerable precision when used to calculate the compressive strength of concrete.

### Concrete strength machine learning model explanation with SHAP

In our study, we employed SHAP as an essential tool for explaining the predictions of the XGBoost machine learning model as it produced the best performance results in terms of R^2^ and RMSE values for concrete strength prediction. SHAP is a powerful, explainable artificial intelligence technique that aids in understanding the intricate relationship between input features and model predictions. It provides valuable insights into which features have the most substantial impact on concrete strength prediction and how they influence the outcomes. By analyzing the SHAP values, we identified the key contributing factors and their degree of influence on the XGBoost model. The visualizations generated by SHAP, including dependency charts and summary plots, helped to uncover non-linear relationships and interactions between features, further enhancing the XGBoost model's interpretability and robustness. With the SHAP method, the XGBoost machine learning model for concrete strength prediction is explained to make its prediction results more transparent and valuable for real-world applications in the construction and engineering industry.

**Figure 13:**
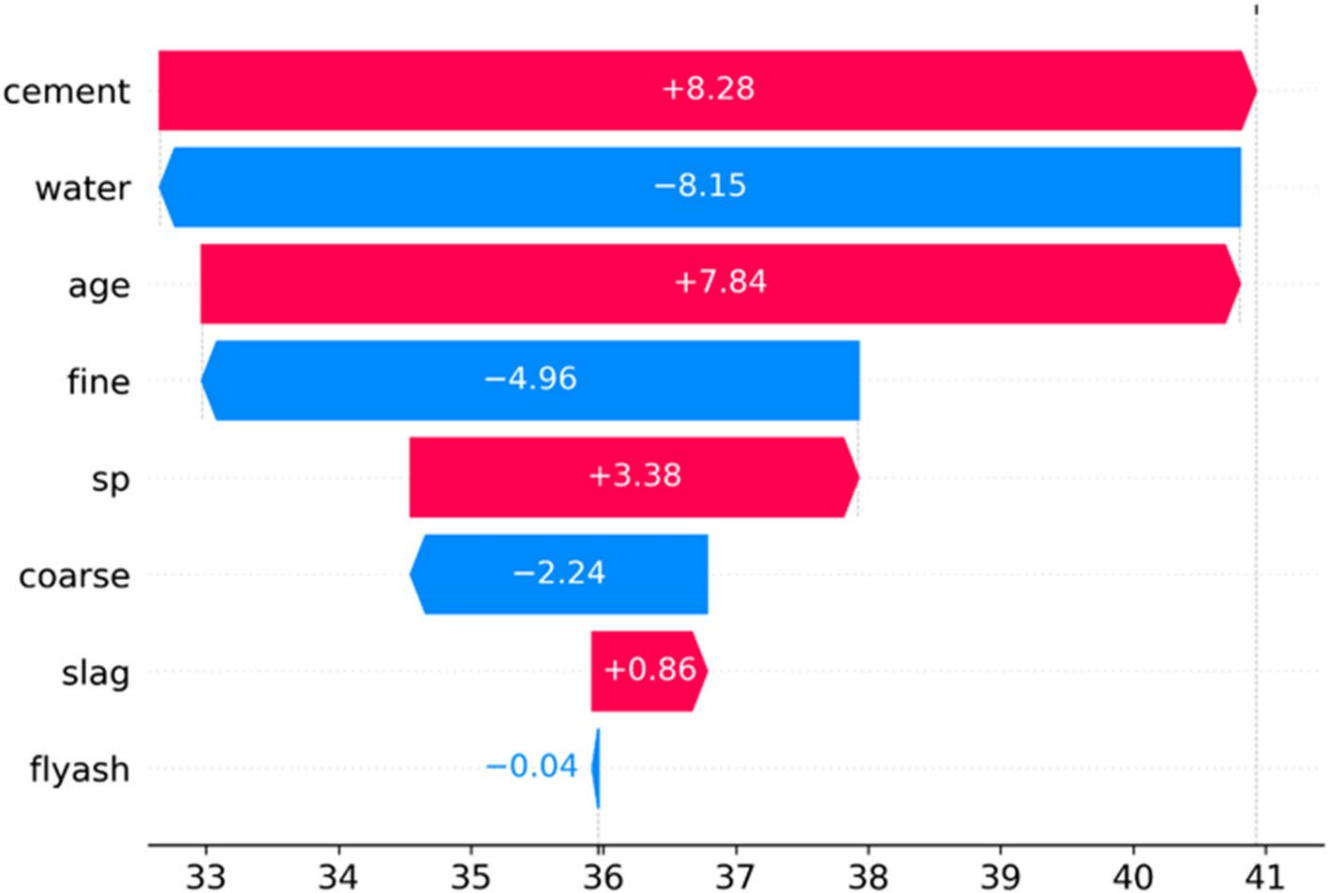
Waterfall plot for studying the impact of different features on the model prediction results.

The cumulative impact of each feature can be depicted in a waterfall chart (Fig. 13), which shows how different features affect the model's output as it changes from the base value—the average model output across the training dataset—to the final predicted value. The probability distribution of compressive strength is plotted from the bottom up, illustrating how the introduction of a specific feature changes the baseline probability from 0 to 100%. The chart uses color coding, with red indicating characteristics that enhance the forecast and blue indicating characteristics that reduce it. For instance, cement increases compressive strength by 80.28%, while water decreases it by 80.15%. The findings show that cement, age, superplasticizer, and slag considerably influence the model's prediction, driving it towards higher values. On the other hand, the water feature has little impact on improving the accuracy of the predictions.

**Figure 14:**
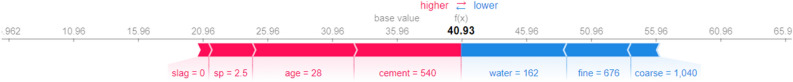
Force plot highlighting the features' optimal values to maximize the model prediction results.

This information is reinforced by the force map in Fig. 14 by emphasizing the features' ideal values to maximize the predicted outcome. The recommended values for the respective components are as follows: to attain a compressive strength equals 40 psi, the values of the input parameters are superplasticizer = 2.5%, age = 28 days, and slag = 0 kg/m^3^; cement = 540 m^3^. However, it should be noted that the prediction result can be further enhanced by adjusting the values of the water, fine aggregate, and coarse aggregate properties. These characteristics are crucial in improving the prediction outcome and provide flexibility in modifying their values to obtain desired outcomes.

**Figure 15:**
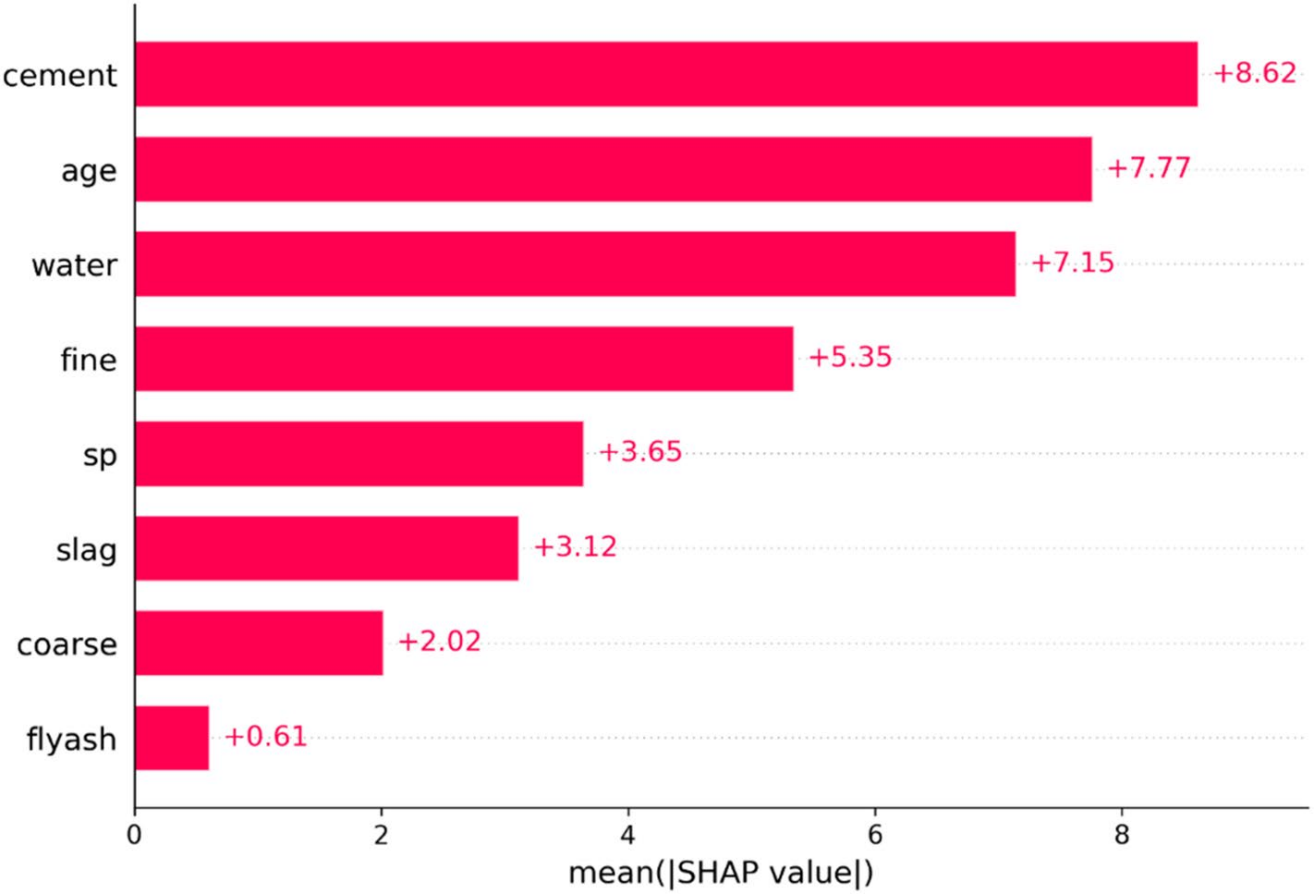
Bar plot of identification of the most model significant features using average SHAP values.

The selection of the most important contributing features to the model predictions is shown in Fig. 15. We guarantee a thorough analysis by determining the average SHAP value across all observations for each feature. The nullification of positive and negative numbers is avoided by averaging the absolute values. The resulting bar plot displays distinct bars for each feature, with cement having the greatest mean SHAP value, suggesting the feature's highest significance level. Notably, either positively or negatively, features with high mean SHAP values have had a considerable impact on the model's predictions. Consequently, these traits significantly impact how the model predicts outcomes.

**Figure 16:**
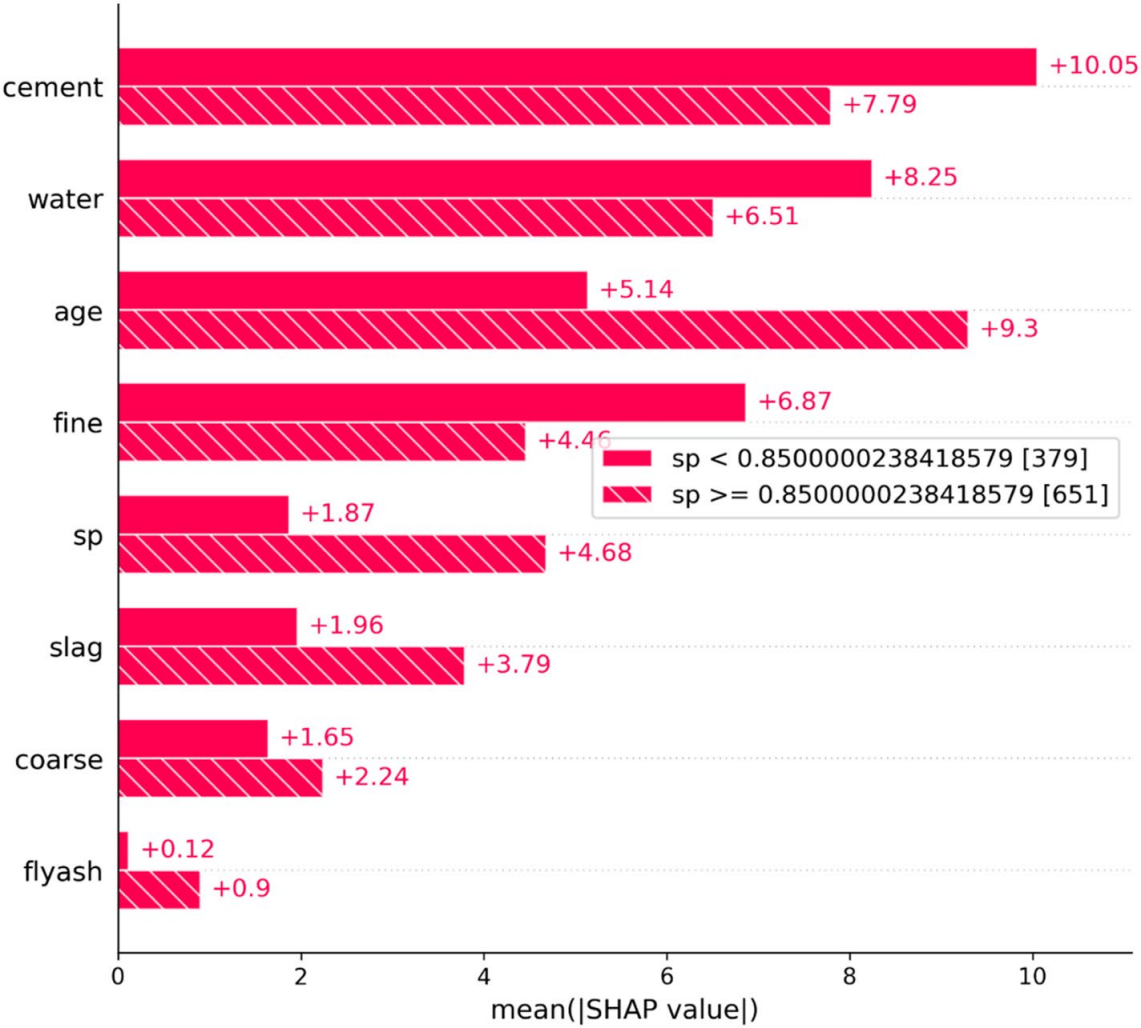
Cohort plot for visualizing the distribution of two groups across different concrete strength prediction features.

Within the dataset, a particular variable leads to the division of data into distinct groups, offering valuable insights into population heterogeneity. As depicted in Fig. 16, our data is effectively segmented into two distinct cohorts. Through automated partitioning, 379 samples are allocated to one cohort, while the remaining 651 samples belong to the second cohort. The optimal threshold for this division is determined to be sp = 0.85. The accompanying bar plot makes it clear that a sample is classified into the cohort where sp ≥ 0.85. This classification is characterized by higher values of age, slag, and coarse features, while exhibiting a lower value for cement.

**Figure 17:**
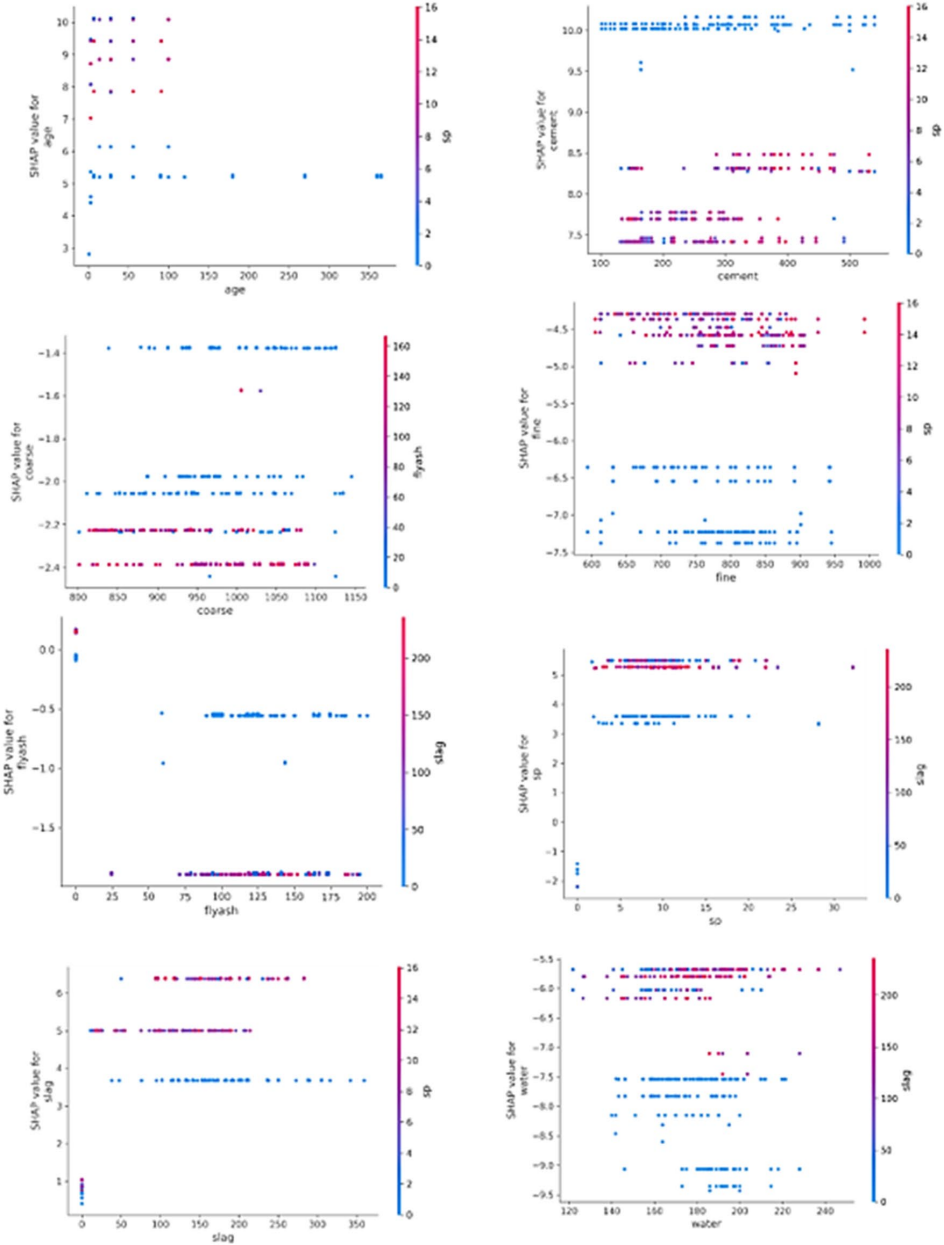
Concrete strength prediction features dependencies.

Figure 17 displays several dependency charts that highlight the conclusions drawn from our model. The dependence plot's dots indicate a single dataset prediction (row). The y-axis designates the appropriate SHAP value, and the x-axis shows the feature value obtained from the features matrix. The SHAP value denotes the degree to which the model's output for a given prediction is affected by knowing the value of a particular feature. The color mapping in the plot represents a second feature that might interact with the plotted feature. For instance, if superplasticizer interacts with cement directly, this will show in the color variations.

From the features dependencies plot in Fig. 17, it is evident that:

- Superplasticizer directly affects only the feature values of cement, age, slag, and fine aggregate in the model's output.
- Fly-ash interacts exclusively with coarse aggregate.
- Slag influences flyash, superplasticizer, and water.

**Figure 18:**
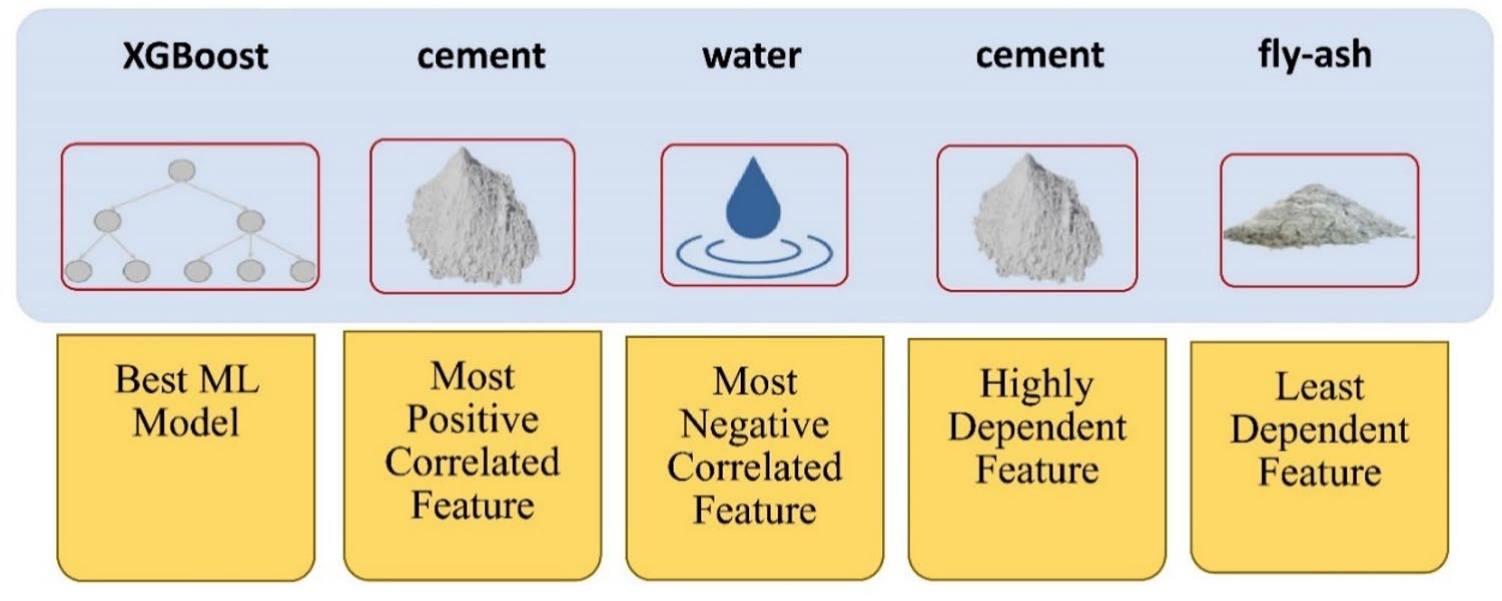
Summary of concrete strength prediction results: machine learning and XAI.

In summary (Fig. 18), it is evident that among all the tested classifiers, XGBoost performs the best with an accuracy of 91%. Consequently, its results were chosen to evaluate the impact of model features on the classifier output. Following explainable AI methodologies, the SHAP method was employed, as it provides comprehensive analysis with supported visual explanations for the classification model. The analysis revealed that cement exhibits the highest positive correlation with concrete strength, while water shows the most significant negative correlation with the compressive strength output. It is noteworthy that the most influential features in predicting concrete strength, ranked in descending order of importance, are cement, age, and water.

Collecting concrete data is a time-consuming and costly process. Samples require extended curing periods, ranging from 28 days for engineering applications to several years for durability studies. Additionally, concrete mixtures and properties are complex to collect and organize systematically. Consequently, much of the publicly available data is relatively small, noisy, and incomplete, often with missing values and imbalanced sample distributions. The values of input features that influence machine learning model predictions are affected by various internal mixture factors, including type, quantity, physical, and chemical properties, which can change over time. External environmental conditions, such as temperature and humidity, also play a role^39^. Variability in concrete mixtures, environmental conditions, and curing processes can introduce uncertainty, while non-standardized testing procedures across different data sources can impact model performance. The selection of input features significantly influences model accuracy. Excluding relevant features or including irrelevant ones can lead to suboptimal predictions. Furthermore, changes in materials, construction techniques, or environmental factors over time may render models developed on historical data less relevant. Complex models may also demand substantial computational resources, making them less practical in certain situations. To address these limitations and potential sources of error, it's important to apply rigorous data preprocessing, validation, and testing procedures, as well as to continually evaluate and update models as new data becomes available.

## Conclusion

In this research paper, we conducted a comparative study to evaluate the performance of eight different machine learning models in predicting concrete strength. The findings suggest that utilizing an artificial neural network with continuous data is suboptimal due to the extended time required for network training. However, the XGBoost model outperforms other established benchmark models and delivers the best results in terms of R^2^ and RMSE. Consequently, we employed the SHAP method, one of the XAI techniques, to analyze the XGBoost classification results and enhance the model's interpretability. The SHAP method provides a comprehensive analysis and visualization of individual features, enabling us to understand how each feature influences the prediction of concrete strength. Additionally, the SHAP method investigates the correlation and dependencies between different features, uncovering the non-linear relationships between features and the model's output. Concrete strength prediction plays a crucial role in real-world engineering and construction scenarios. These predictions are used to assess whether concrete meets required strength standards, thereby helping to prevent issues like structural failures. Engineers and architects utilize concrete strength predictions to select the appropriate mix design for specific applications and environmental conditions, determine the size and quantity of concrete reinforcements needed for various structural elements, ensure the safety of buildings and infrastructure, reduce overdesign, leading to cost savings in materials and construction, and contribute to the longevity and durability of structures. Optimized concrete mix designs based on predictions can also help reduce the environmental impact of construction by minimizing the use of raw materials and reducing waste production. In the future research directions, we plan to expand our analysis by considering additional features for concrete strength prediction, including the cement fermentation period and various concrete’s mechanical, physical, and chemical properties. This expansion will help to enhance the analysis and prediction capabilities of different machine learning models.

## Data Availability

Data is available online at UC Irvine Machine Learning Repository (https://archive.ics.uci.edu/dataset/165/concrete+compressive+strength).
